# Electrospun Composite Liquid Crystal Elastomer Fibers

**DOI:** 10.3390/ma11030393

**Published:** 2018-03-07

**Authors:** Anshul Sharma, Jan P. F. Lagerwall

**Affiliations:** Physics and Materials Science Research Unit, University of Luxembourg, 162 A Avenue de la Faïencerie, 1511 Luxembourg, Luxembourg

**Keywords:** liquid crystal elastomers, electrospinning, fibers, nematic, core-sheath, actuators, polyvinylpyrrolidone, polylactic acid

## Abstract

We present a robust method to prepare thin oriented nematic liquid crystalline elastomer-polymer (LCE-polymer) core-sheath fibers. An electrospinning setup is utilized to spin a single solution of photo-crosslinkable low molecular weight reactive mesogens and a support polymer to form the coaxial LCE-polymer fibers, where the support polymer forms the sheath via in situ phase separation as the solvent evaporates. We discuss the effect of phase separation and compare two different sheath polymers (polyvinylpyrrolidone and polylactic acid), investigating optical and morphological properties of obtained fibers, as well as the shape changes upon heating. The current fibers show only irreversible contraction, the relaxation most likely being hindered by the presence of the passive sheath polymer, increasing in stiffness on cooling. If the sheath polymer can be removed while keeping the LCE core intact, we expect LCE fibers produced in this way to have potential to be used as actuators, for instance in soft robotics and responsive textiles.

## 1. Introduction

Liquid crystal elastomers (LCEs) are a specific class of lightly cross-linked elastomeric materials that combine the properties of self-organization and responsiveness of liquid crystals (LCs) with the entropy elasticity of a polymeric rubber [1,2,3]. This gives them the ability to strongly change their shape in response to any stimulus that affects the degree of LC order, such as heating. LCEs are best employed in several applications such as artificial muscles [4], scaffolds in tissue regeneration [5], mirrorless lasing [6], microswimmers [7], artificial cilia [8], mimicking seedpod opening [9] or the closing of a Venus flytrap [10], light fuelled wave machine [11] and many more [1,2,3]. Typically, an LCE network consists of either side chain or main chain LC monomers chemically or physically bonded by a weakly cross-linked network that maintains a predefined molecular orientation. Since the introduction of the LCE concept by de Gennes [12], several methodologies to control the molecular orientation and to tailor the response to various external stimuli, thus resulting in reversible macroscopic deformation, have been reported [1,2,3,13].

In most cases, an LCE precursor mixture is filled between two glass plates that are treated to impose a predefined alignment of the liquid crystal-forming molecules (mesogens), followed by polymerization to generate oriented anisotropic elastomeric films. After removal from the substrates, the resulting film can be explored as an actuator, showing various movements or shape changes depending on thermal, mechanical, chemical or electrical stimuli. Recently, there has been significant work done to incorporate liquid crystal (LCs) into polymer fibers using electrospinning [14,15,16,17,18] or air-brushing techniques [19]. Reyes et al. employed coaxial electrospinning as a versatile technique to obtain, e.g., fiber-based soft gas sensors with a low molar mass LC as core material enclosed by a cylindrical polymer sheath, that can function as non-electronic artificial nose to detect organic vapors at room temperature [20]. The team of West and Jakli pioneered the technique of electrospinning coaxial LC-polymer fibers from a single solution, obtaining impressive results thanks to in situ phase separation between polymer and low molar mass LC as the solvent evaporates [15,18]. Many groups have reported fibers from lyotropic [21] and thermotropic liquid crystal polymers (LCPs) [22,23,24] including commercial LCPs such as Vectra and Kevlar using either electro or melt spinning [25,26,27]. However, since the first report by Naciri et al. on LCE fibers obtained by drawing from a polymer melt [28], only a few researchers have reported the formation of oriented LCE fibers that show thermally induced shape changes, using techniques such as melt extrusion [29] and wet spinning assisted by a microfluidic setup [30,31,32].

As LCs are known to align in an external electric or magnetic field, this property of LCs can be explored via electrospinning to generate continuous oriented fibers. However, the low molecular weight of LC monomers makes it impossible to spin LC monomers directly. Different strategies can be devised to overcome this problem [33]. Wu et al. used blends of polysiloxane-based side chain cholesteric LCPs and polyethylene oxide to obtain a spinnable solution and fibers [34]. Yao et al. recently showed that blends of reactive LC monomer and polymer can be used to obtain oriented core-sheath fibers via phase-separation electrospinning [35]. Electrospinning of reactive mesogens to generate actuating LCE fibers still remains an important challenge. Even in recent work, less controlled methods for forming the LCE fibers are used, relatively thick fibers forming by dipping the tip of a metal bar into a viscous precursor solution and quickly pulling away, with simultaneous UV irradiation [36].

Here we apply electrospinning with in situ phase separation as a method to obtain oriented core-sheath fibers with an LCE core formed by a reactive mesogen mixture (RMM) that spontaneously separates from an added sheath polymer during spinning. We compared two polymers available with high molar mass for the supporting sheath formation, polyvinylpolypyrrolidone (PVP) and polylactic acid (PLA). The RMM is a suitable precursor mixture consisting of reactive mesogen (M), crosslinking agent (CL) and a photoinitiator (PI). This is co-dissolved together with the PVP or PLA in a suitable solvent or solvent mixture, and photo-polymerized after fiber formation to form the LCE network and fix the molecular orientation. We characterize the fibers formed with each type of supporting sheath polymer under varying conditions, and we discuss the different degrees of actuation, fiber morphology, and LC order parameter changes upon heating and cooling.

## 2. Results and Discussion

**Figure 1:**
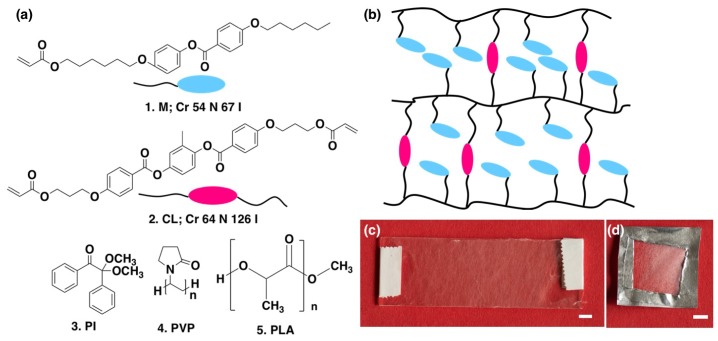
Fiber composition: (**a**) Chemical structure of: 1. reactive monomer (M); 2. cross-linker (CL); 3. photo-initiator (PI); 4. polyvinylpolypyrrolidone (PVP) and 5. polylactic acid (PLA); (**b**) Schematic showing LCE network formed by reactive monomer and cross-linker (the smectic-like organization is only due to the simplified drawing; the real LCE should be nematic); (**c**) LCE-PVP fibers spun on glass and polymerized at 55 ${}^{\circ }$C and (**d**) the same type of fibers spun free-hanging on a frame (scale bars: 5 mm).

Our goal is to prepare fibers from nematic LCEs in a reproducible way using the electrospinning method. In order to achieve this, we used commercially available LC monomers that exhibit only a nematic mesophase as starting materials and that have been used previously in LCE studies. The structures of the RMM components, as well as of the sheath polymers and of the photoinitiator (PI), are shown in Figure 1.

As has been reported previously [37] that, in a side chain LC polymers and elastomers mesogen network, end-on side chain mesogen units can have a parallel orientation to the polymer backbone depending on alkyl spacer length, which in our system is 6 in M (Figure 1a). The monomer design results in an “end-on” side-chain LCE network (Figure 1b), i.e., the mesogens (M as well as CL) are attached to the polymeric backbone with parallel orientation. While main-chain and “side-on” side-chain LCEs are expected to yield actuation, they also impose a more involved chemistry, requiring components such as thiols that are awkward to handle safely or reactive mesogens that are not commercially available. As a test of the concept, we thus settled with an end-on side-chain LCE structure. First spinning conditions are optimized for both LCE-PVP and LCE-PLA to obtain continuous cylindrical fibers and then the fibers are characterized by polarizing optical microscopy (POM) and scanning electron microscopy (SEM), before and after heating, for investigating the optical properties, fiber morphology, and actuation behavior.

### 2.1. Spinning Conditions and Polymerization

**Figure 2:**
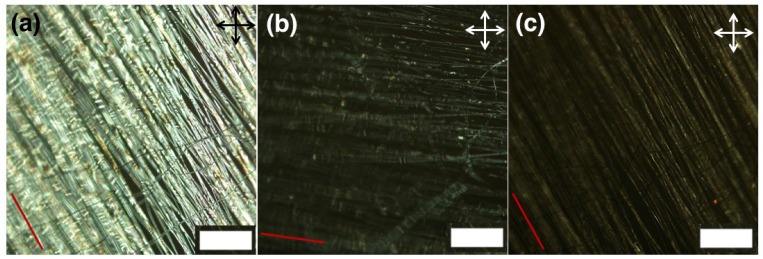
POM of LCE-PVP fibers spun aligned on glass (polymerized at 55 ${}^{\circ }$C), (**a**) between crossed polarizer and analyzer, with average fiber orientation (red line) roughly at 45${}^{\circ }$ to the polarizer and (**b**) roughly parallel to the polarizer; In (**c**) the fibers have the original orientation, as in (**a**), but they are heated to 72.5 ${}^{\circ }$C, at which the core turns isotropic, leading to strong reduction in birefringence. The red line represents the average fiber orientation of fibers. (scale bars: 200 μm).

**Figure 3:**
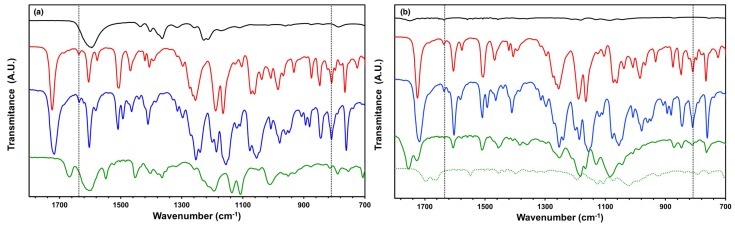
IR spectra of (**a**) pure PVP (black); M (red); CL (blue) and LCE-PVP fibers polymerized at 55 ${}^{\circ }$C (green continuous) and (**b**) pure PLA (black); M (red); CL (blue); LCE-PLA fibers polymerized at 55 ${}^{\circ }$C (green continuous) and CL-PLA fibers polymerized at room temperature (green dashed). The dashed line highlight the regimes of the C=C stretch (1635 cm${}^{-1}$) and C=H bend (810 cm${}^{-1}$) vibrations, respectively.

It is well known that spinning parameters (such as solution concentration, spinning voltage, working distance, and solution feeding rate) and spinning conditions (temperature and relative humidity) have a great effect on the morphology of fibers (see [33] and references therein). First we established if the phase separation electrospinning method can be used to make core-sheath LCE-polymer fibers. As our previous work is based on PVP as sheath polymer, and as Wang et al. successfully spun low molar mass LC-PVP fibers using the phase separation method [18], we decided to test PVP as an outer sheath. The in situ phase-separation electrospinning method is also used by Yao et al. [35] to generate core-sheath fibers with a reactive mesogen core and PMMA or polyamide 6 sheath. For our fibers we prepared a spinning solution with the relative mass fractions 11:10:79 of PVP, RMM and solvent mixture [ethanol:chloroform, 8:2 (*v:v*)], respectively, which is spun at spinning voltage 5–8 kV and collection distance 12 cm. After spinning, the obtained fibers are transferred to a hot plate at 55 ${}^{\circ }$C so that the reactive reactive precursors in the core are in nematic phase and exposed to UV light for 30 min to initiate the photo-polymerization to obtain LCE-PVP fibers (collected on the glass, Figure 2). The polymerization of the reactive precursors in the core into LCE is confirmed by IR spectroscopy. Figure 3a shows the IR spectra of pure PVP, monomer, cross-linker and LCE-PVP fibers cured at 55 ${}^{\circ }$C, respectively. The IR absorption bands at 1635 cm${}^{-1}$ (C=C stretch) and 810 cm${}^{-1}$ (C=H bend) are absent in the LCE-PVP spectrum, indicating completion of the polymerization reaction [38].

Additionally, we spun fibers with PLA sheath, again inspired by the work of West and Jakli, demonstrating that PLA is suitable for encapsulating low molar mass LCs [15,18] and thus established suitable spinning parameters for both pure PLA and LCE-PLA fibers. The concentration of PLA in a chloroform and acetone solvent mixture [3:1 (*v:v*)] is varied between 5, 8, 9 and 10.3 wt. %, keeping the spinning voltage in the range 7–9 kV and the collection distance at 12–17 cm. Before electrospinning, an RMM-PLA solution to make LCE-PLA fibers, we decided to test the spinning conditions for PLA and a single reactive mesogen (CL) in the same solvent mixture. We made three different compositions (mixture 1, mixture 2 and mixture 3), as summarized in Table 1, which are then electrospun with a spinning voltage of 7–8 kV and collection distance of 17 cm, followed by UV curing at room temperature.

For the LCE-PLA case, we chose a spinning solution with mass ratio 10.1:10.5:79.4 (similar to mixture 2) of PLA, RMM and solvent mixture, respectively. The obtained fibers are photo-crosslinked for 30 min at 55 ${}^{\circ }$C to ensure that the core is transformed into an LCE. Similar to the case of the LCE-PVP fibers, the polymerization of the reactive core in the CL-PLA and the LCE-PLA fibers is confirmed by IR spectroscopy. Figure 3b shows the IR spectra of pure PLA, monomer, cross-linker, CL-PLA fibers cured at room temperature and LCE-PLA fibers cured at 55 ${}^{\circ }$C. For CL-PLA fibers the IR absorption bands at 1635 cm${}^{-1}$ (C=C stretch) and 810 cm${}^{-1}$ (C=H bend) are absent but for LCE-PLA fibers (cured at room temperature or at 55 ${}^{\circ }$C) there is still unreacted acrylate left.

### 2.2. Characterization of LCE-PVP Fibers

The obtained LCE-PVP fibers are firstly observed under the POM. The resultant fibers are smooth and highly birefringent (bright between crossed polarizers) as shown in Figure 2 and show extinction when the fiber axis is parallel or perpendicular to the polarizer Figure 2b. This indicates that in the LCE-PVP fibers the mesogens are aligned along the fiber axis. According to the standard experiments of LCE actuation (see [1,2,3] and references therein), on heating a well-oriented LCE sample to the nematic-isotropic transition we should expect a reversible macroscopic contraction along the original director since the stretching of the polymer chain along the director disappears when the nematic order is lost. For the end-on side-chain, LCE architecture probed here the effect is less straightforward than for main-chain and side-on side-chain LCEs, but it should be noted that the schematic in Figure 1b is somewhat misleading since it does not take into account the 3D extension of the polymer network and the fact that the LCE is nematic. We wanted to test if contraction is indeed observed in our well-oriented fibers.

**Figure 4:**
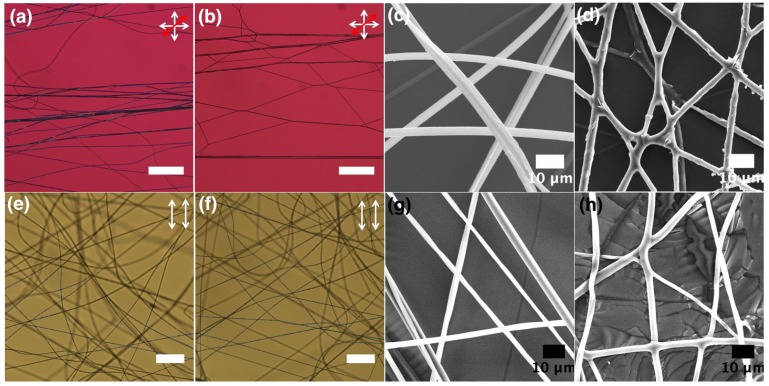
POM and corresponding SEM image of: (**a**,**c**) LCE-PVP fibers at 20 ${}^{\circ }$C; (**b**,**d**) LCE-PVP fibers in isotropic phase at 72.5 ${}^{\circ }$C; (**e**,**g**) pure PVP fibers at 20 ${}^{\circ }$C and; (**d**,**h**), after heating to 75 ${}^{\circ }$C. Images (**a**,**c**) are with red wave plate between crossed polarizers (scale bars: 200 μm.) The white double-ended arrows depict the orientation of polarizer and analyzer, respectively, and the red double-headed arrow in (**a**,**b**) shows the direction of optic axis of a first-order λ plate inserted in the light path.

In order to observe actuation during the phase transition from nematic to the isotropic phase, an LCE-PVP fiber sample is heated in a hot stage at the rate of 1 ${}^{\circ }$C per minute to the isotropic phase. As shown in Figure 2c, a birefringence loss is observed but no movement of fibers is detected. This could be attributed to the fact that the fibers are spun on the glass surface and are unable to move due to being fixed on a substrate. It is important to check a sample with free-hanging fibers, that have no hindrance from the substrate. In order to test this, we designed a flexible frame made out of the inert fluoropolymer Bytac, to which free-hanging LCE-PVP fibers, spun between two parallel grounded electrodes, are carefully transferred (Figure 1d). This framed free-hanging sample is studied by POM during heating to test for any deformation induced by the phase transition from nematic to the isotropic in the LCE core. The sheath polymer is not cross-linked and is initially solid as it is below the glass transition temperature. On heating from 20 ${}^{\circ }$C (Figure 4a), we noted that the birefringence disappeared and the fibers started merging once the temperature reached 72.5 ${}^{\circ }$C, corresponding to the clearing point. As shown in the POM image obtained after heating to the isotropic phase, the LCE-PVP fibers formed a web-like network (Figure 4b). As a reference, we also spun free-hanging pure PVP fibers in a similar frame. When these fibers are heated to the same temperature no web-like network is formed (Figure 4f).

**Figure 5:**
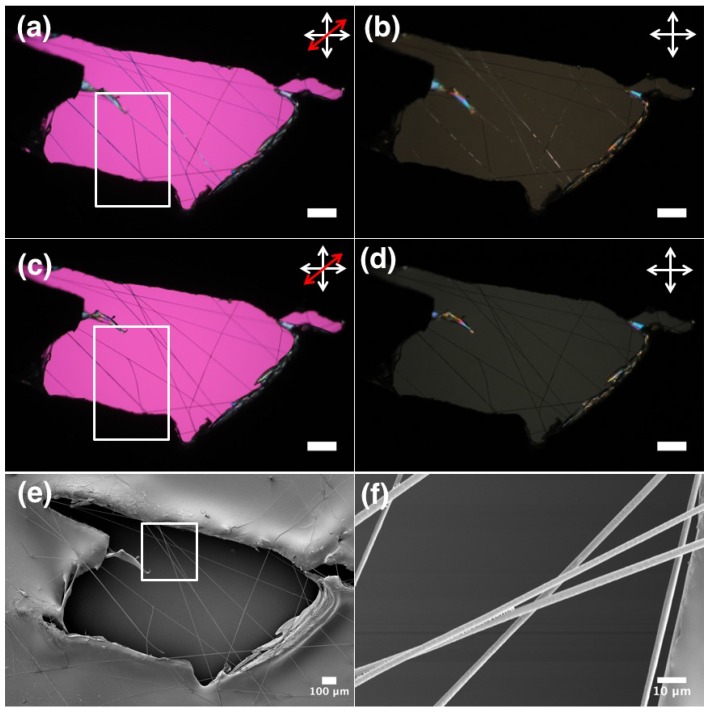
POM image of LCE-PVP fibers (**a**,**b**) at 20${}^{\circ }$ and (**c**,**d**) in the isotropic phase at 70 ${}^{\circ }$C; (**e**,**f**) SEM images of the same sample heated to 75 ${}^{\circ }$C. Images a and b are with red wave plate between crossed polarizers (scale bars for images (**a**–**d**): 200 μm). The white double-ended arrows depict the orientation of polarizer and analyzer, respectively, and the red double-headed arrow in (**a**,**c**) shows the direction of optic axis of a first-order λ plate inserted in the light path.

Both LCE-PVP and pure PVP fiber samples are observed with SEM for morphological changes before and after heat exposure. The average diameter of as-spun PVP fibers is 2.6 ± 0.9 μm, which on heating to 75 ${}^{\circ }$C changed only slightly to 2.5 ± 0.5 μm. However, the average diameter of LCE-PVP fibers (polymerized at 55 ${}^{\circ }$C) increased from 2.7 ± 0.6 μm to 3.0 ± 0.7 μm, which could be due to merging of fibers on heating above the phase transition temperature. We are quite intrigued by these observations and wanted to further observe not just a few fibers but a whole sample area during heating, hence a small free-hanging sample is carefully fabricated and observed under POM during the phase transition (Video S1, Figure 5). On heating, the first detection of fiber movement is observed at about 44 ${}^{\circ }$C, with the strongest fiber response occurring around 61 ${}^{\circ }$C. The fiber cores went isotropic around 75 ${}^{\circ }$C (Figure 5c,d). On cooling the LCE-PVP fibers, the nematic state comes back at around 68 ${}^{\circ }$C but no reverse movement is observed, as once fibers are merged together it is difficult to separate them.

**Figure 6:**
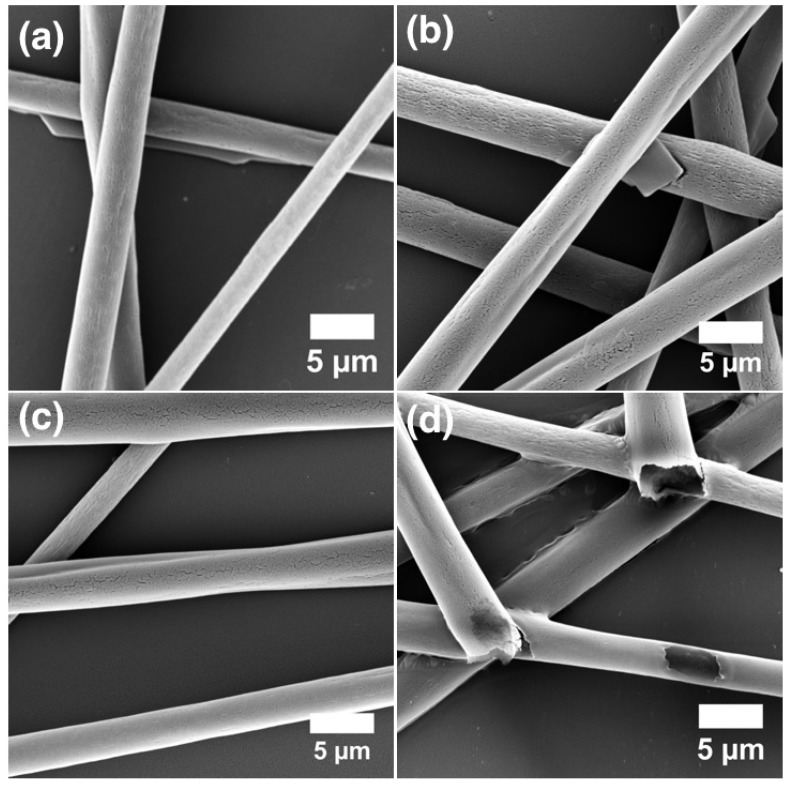
SEM image of LCE-PVP fibers: (**a**) without UV and heat exposure; (**b**) heated to 55 ${}^{\circ }$C; (**c**) after UV exposure at 55 ${}^{\circ }$; and (**d**) after UV exposure at 55 ${}^{\circ }$C and subsequent heating to 75 ${}^{\circ }$C, above the clearing point of the LCE.

The heated LCE-PVP fiber sample is investigated by SEM to get a better understanding of its surface morphology after actuation. Interestingly, these free-hanging fibers showed a teething type morphology in the areas surrounding points where fibers have merged together (Figure 5e,f). In order to better understand this morphological change in the LCE-PVP fibers during heating to the isotropic phase, it is important to know if this effect is due to heat and UV exposure during polymerization or is as a result of the LCE undergoing a phase transition on heating. We thus investigated the morphologies of LCE-PVP fibers at different stages by SEM, comparing as-formed LCE-PVP fibers (with no UV and heat exposure), fibers heated to 55 ${}^{\circ }$C, fibers UV exposed at 55 ${}^{\circ }$C and fibers UV exposed at 55 ${}^{\circ }$C and then heated to 75 ${}^{\circ }$C (Figure 6). To simplify the SEM investigations, these fibers are spun directly on a substrate rather than free-hanging.

The average diameter of the LCE-PVP fibers without any UV and heat exposure is 3.2 ± 0.6 μm (Figure 6a), while the diameter after heating to 55 ${}^{\circ }$C is possibly slightly reduced to 3.1 ± 0.4 μm (Figure 6b), although the difference is smaller than the error margin. No major change in fiber morphology is observed between these two sample types. However on UV-induced polymerization at 55 ${}^{\circ }$C, the diameter of the LCE-PVP fibers is reduced more significantly to 2.6 ± 0.7 μm (Figure 6c), yet still with no clear change in morphology. The reduction of diameter may be due to the shrinkage during polymerization of the core. After the UV-polymerized fibers are subjected to the final heating to 75 ${}^{\circ }$C, the diameter of fibers appeared to have increased to 3.3 ± 0.7 μm (Figure 6d). At least one fiber, of which the cross section is seen, appears to be somewhat flattened. It may thus be that the heating has softened the fiber, allowing the interaction with the substrate to promote spreading and flattening, yielding an apparent diameter increase in the SEM images, taken orthogonally to the substrate. No sign of the teething-like sheath morphology, as seen in the free-hanging sample upon heating, could be seen in these substrate-deposited fibers.

We can conclude that PVP can be used as supporting polymer to produce LCE core-PVP sheath fibers. However, the hygroscopic nature of PVP makes it impractical to work with in many respects. For instance, the relative humidity of the spinning atmosphere greatly affects the morphology of PVP and LC-PVP fibers [18,20]. We thus decided to explore also the more hydrophobic PLA as the supporting polymer, forming the sheath around the LCE precursor core, as no humidity effect on the morphology of LC-PLA is reported [18].

### 2.3. Characterization of PLA, CL-PLA and LCE-PLA Fibers

**Figure 7:**
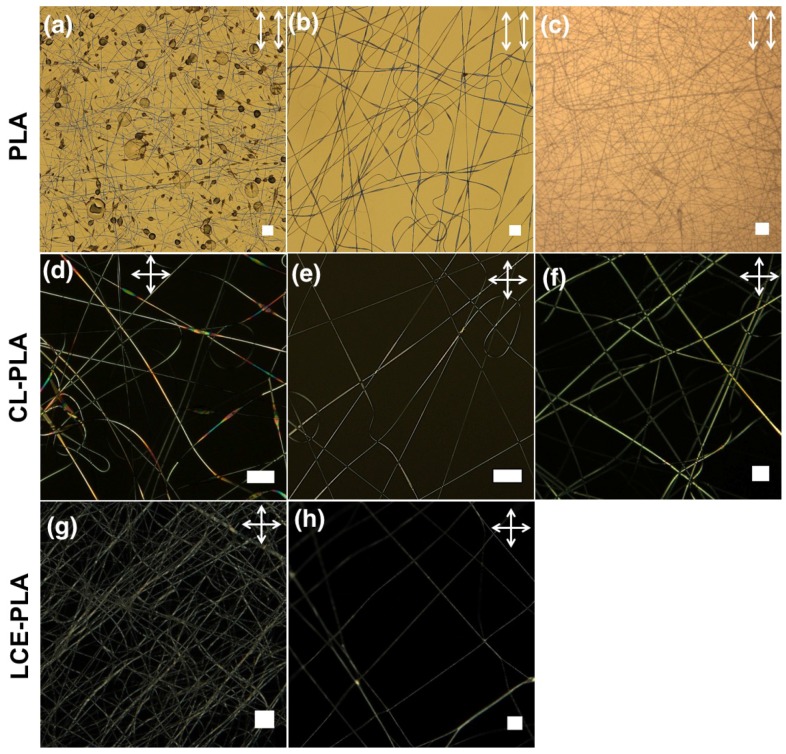
POM of pure PLA fibers spun with a PLA concentration of (**a**) 5; (**b**) 8 and (**c**) 9 wt. % respectively; CL-PLA fibers spun from different solutions: (**d**) mixture 1; (**e**) mixture 2 and (**f**) mixture 3; (**g**) LCE-PLA fibers spun randomly on glass and (**h**) free-hanging (both are UV cured at 55 ${}^{\circ }$C). (scale bars: 200 μm.) The white double-ended arrows depict the orientation of polarizer and analyzer, respectively.

PLA, CL-PLA and LCE-PLA fibers are characterized by POM and SEM for their optical and morphological characteristics, respectively. For pure PLA fibers, a continuous cylindrical morphology without beading is observed with 9 wt. % PLA at a spinning voltage of 7 kV and collection distance of 15 cm (Figure 7c). As shown in the POM images (Figure 7d–f), for CL-PLA, uniform fibers without beading are obtained from the mixture, 2 (Figure 7e) and 3 (Figure 7f) only.

**Figure 8:**
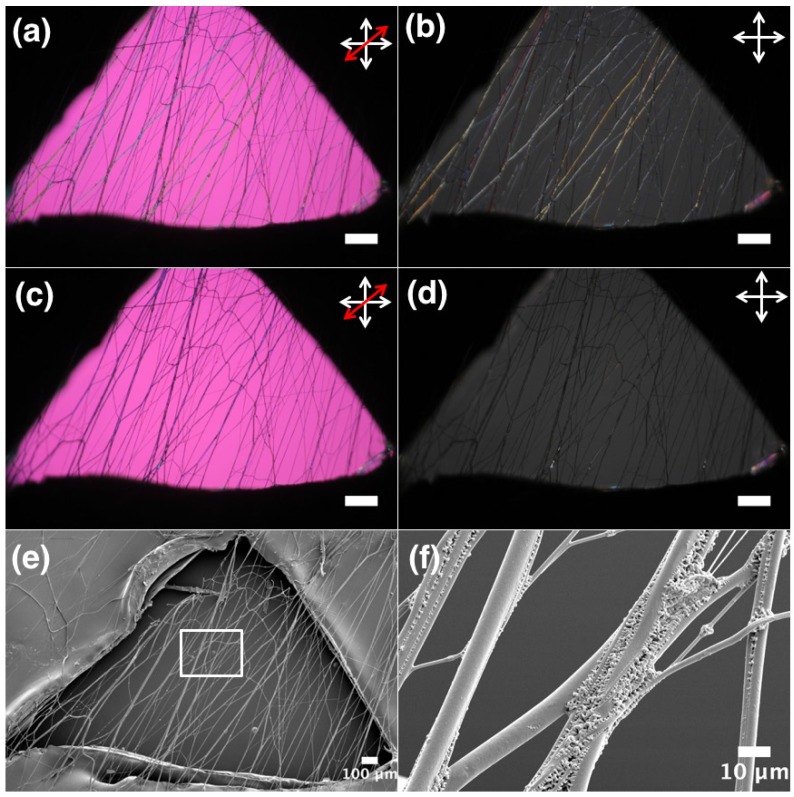
POM image between crossed polarizers: (**a**,**b**) PLA + LCE fibers at 20 ${}^{\circ }$C; (**c**,**d**) PLA + LCE fibers: at isotropic phase at 70 ${}^{\circ }$C; and (**e**,**f**) SEM image of same sample heated to 75 ${}^{\circ }$C above phase transition of LCE. Images a and c are with red wave plate between crossed polarizers (scale: 200 μm). The white double-ended arrows depict the orientation of polarizer and analyzer, respectively, and the red double-headed arrow in (**a**,**c**) shows the direction of optic axis of a first-order λ plate inserted in the light path.

For LCE-PLA fibers, as shown in Figure 7 we obtained smooth fibers with random orientation (Figure 7g) as well as with uniform alignment (Figure 7h). A free-hanging sample of as-spun and polymerized LCE-PLA fibers are observed in POM during the LCE clearing transition (Video S2, Figure 8). At room temperature birefringent fibers are observed (Figure 8a,b) and on heating some movement or deformation of fibers started at about 56 ${}^{\circ }$C, with more distinct contraction and some fiber merging around 64 ${}^{\circ }$C. The fiber core goes isotropic around 74 ${}^{\circ }$C (Figure 8c,d). On cooling the LCE-PLA fibers, the nematic state comes back at around 68 ${}^{\circ }$C but no reverse movement is observed. Once fibers are merged together it is difficult to separate them. When the same LCE-PLA fiber sample is checked for morphology with SEM, we again detected a teething type morphology around the areas where fibers are merged together, sometimes forming larger bundles (Figure 8e,f).

**Figure 9:**
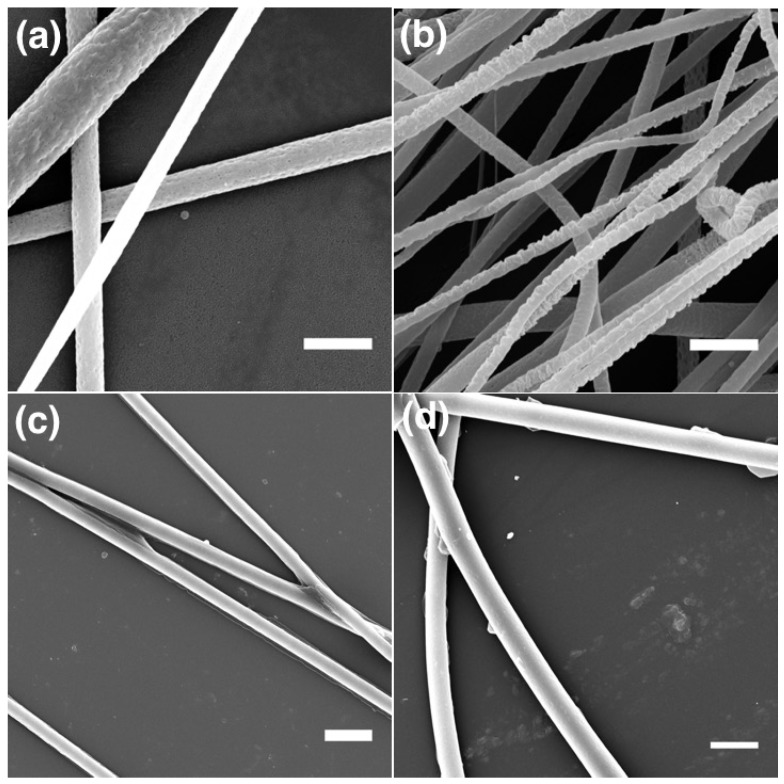
SEM of (**a**) pure PLA fibers (spun from a 9 wt. % solution) without heat treatment; (**b**) the same type of fibers after heating to 80 ${}^{\circ }$C; (**c**) CL-PLA fibers (spun from mixture 2) after UV curing at room temperature and subsequent heating to 55 ${}^{\circ }$C and (**d**) the same type of fibers after UV curing at room temperature and subsequent heating to 80 ${}^{\circ }$C. (scale bars: 10 μm.)

In order to understand the effect of the core on the sheath morphology, we investigated pure PLA and core-sheath CL-PLA fibers by SEM, as shown in Figure 9. We checked the morphology of pure PLA fibers (spun from a 9 wt. % solution) before (Figure 9a) and after (Figure 9b) heat exposure. The average diameter of pure PLA fibers is measured to be 3.6 ± 0.7 μm. On heating the pure PLA fibers to 80 ${}^{\circ }$C, the diameter is reduced to 2.1 ± 0.4 μm and wrinkling of the fiber surface is observed (Figure 9b). The latter phenomenon may be attributed to the melting of the PLA and the resulting viscous polymeric liquid with cylindrical shape trying to reduce its surface area via a Rayleigh instability that, if allowed to continue, would break the fibers into droplets. In contrast, on heating CL-PLA fibers (with cross-linked core) to 55 ${}^{\circ }$C (Figure 9c) and 80 ${}^{\circ }$C (Figure 9d), no such wrinkling is seen, indicating that the cross-linked stiff core kept the fiber stable, despite the liquefied PLA sheath. The average fiber diameter of CL-PLA fibers heated to 55 ${}^{\circ }$C and 80 ${}^{\circ }$C is estimated to be 2.9 ± 0.3 μm and 2.6 ± 0.4 μm, respectively.

**Figure 10:**
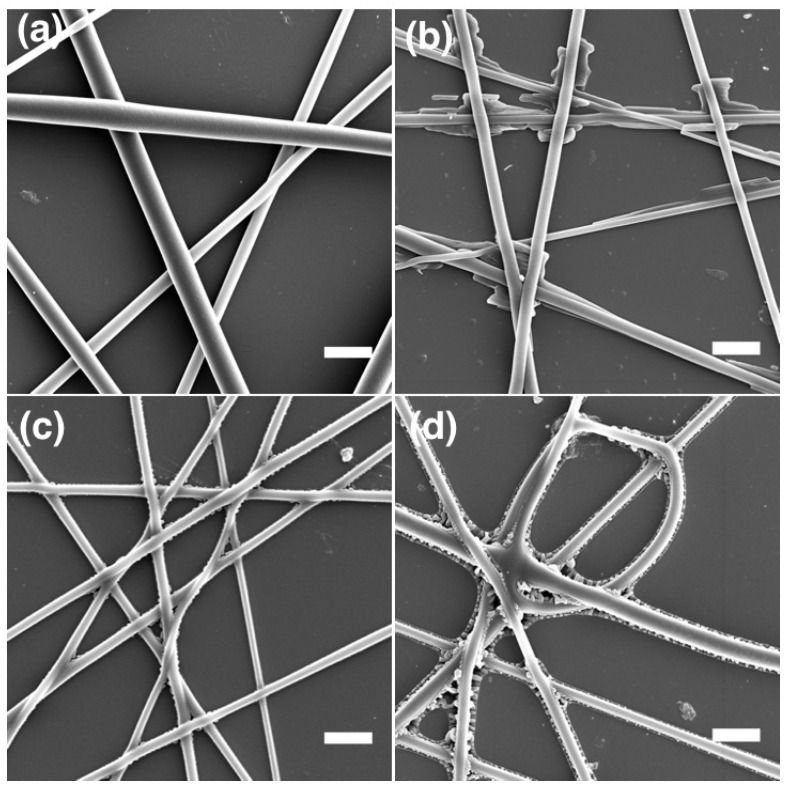
SEM images of LCE-PLA fibers (**a**) without UV and heat exposure; (**b**) heated to 55 ${}^{\circ }$C; (**c**) after UV exposure at 55 ${}^{\circ }$C and (**d**) after UV exposure at 55 ${}^{\circ }$C and subsequent heating to 75 ${}^{\circ }$C, above the clearing transition of the LCE. (scale bars: 10 μm).

We further investigated the morphology of LCE-PLA fibers at different stages of production and after heating, taking SEM images (Figure 10) of as-formed fibers (with no UV and heat exposure), fibers heated to 55 ${}^{\circ }$C, fibers UV exposed at 55 ${}^{\circ }$C and fibers UV exposed at 55 ${}^{\circ }$C and subsequently heated to 75 ${}^{\circ }$C (as shown in Figure 10).

The average diameter of LCE-PLA fibers without any UV and heat exposure as shown in Figure 10a is 2.6 ± 0.54 μm, while their diameter after heating to 55 ${}^{\circ }$C is slightly reduced to 2.4 ± 0.6 μm (Figure 10b). Here we did observe teething along the length of fiber, although the fibers are deposited on a substrate. Upon polymerization (UV exposure and heat) at 55 ${}^{\circ }$C the average diameter is significantly reduced to 1.4 ± 0.3 μm (Figure 10c). We again attribute this shrinkage to the shrinkage of the core upon polymerization. A certain degradation of the sheath is also observed seen in some fiber parts. When fibers that had been UV-polymerized at 55 ${}^{\circ }$C are subsequently heated to 75 ${}^{\circ }$C, i.e., the clearing transition, the fiber diameter again appeared to increase, similar to the case of LCE-PVP fibers, to 2.5 ± 0.7 μm (Figure 10d), suggesting the softening of the PLA forming the sheath, leading to some fiber collapsing due to the capillary forces arising from contact with the substrate. In some areas, merging of fibers and teething at the surface are also observed.

On comparing the effect of heat and UV exposure between LCE-PVP (Figure 6) and LCE-PLA (Figure 10) fibers, we note that the diameter of fibers is reduced upon polymerization and increases on heating above the clearing transition in both the cases. As the sheath gets soften on heating and the LCE core contracts the diameter of fibers has to increase due to volume conservation. The change in morphology is more pronounced in LCE-PLA fibers and we assume that this is related to the difference in glass transition temperature ($T_{g}$) of the two polymers. For pure PVP, $T_{g}\gg 120$ ${}^{\circ }$C and goes to 180 ${}^{\circ }$C for high molecular weight PVP [39], whereas for pure PLA have $T_{g}\approx$ 55–63${}^{\circ }$ [40]. As it is well known that in a polymer, additives act as a plasticizer that lowers $T_{g}$, any contaminant affects the thermal and mechanical properties. This effect is more pronounced when these additives are reactive monomers and oligomers and also depends on the size and flexibility of the additives [41]. A stiff additive has less effect on depression of $T_{g}$ of the polymer, whereas a flexible or elastic additive can greatly lower it [41].

In case of phase separated electrospun low molar mass LC-PLA fibers, the lowering of the glass transition temperature has been previously reported [15], although the effect on morphology is not discussed. Yao et al. have further studied the effect of depression of $T_{g}$ in electrospun fibers of reactive mesogens and polymer (PMMA and polyamide 6, respectively), produced via in situ phase separation during spinning [35]. They observed that in order to have a sufficient phase separation and overcome the effect of reactive mesogen acting as a plasticizer, a certain threshold percentage of reactive mesogen in the spinning solution is required. However, they have one additive with constant size and we have a multicomponent reactive system, which further complicates the situation.

**Figure 11:**
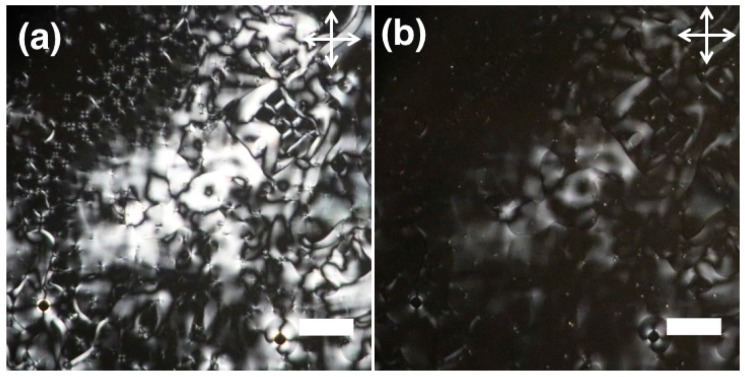
POM image of LCE film polymerized at 55 ${}^{\circ }$C between untreated glass slide and cover slip (crossed polarizers): (**a**) At 20 ${}^{\circ }$C and (**b**) At 115.8 ${}^{\circ }$C at isotropic phase. (scale: 200 μm).

We also looked at the phase transition in an LCE film made out of a reactive mesogen mixture with the same composition as in the RMM-polymer solutions used for our fibers, finding a clearing temperature of about 116 ${}^{\circ }$C (Figure 11). As the clearing temperatures of the LCE film and the LCE-polymer fibers are drastically different, this further indicates that there is a loss of reactive mesogens from the core during fiber production, that remains within the sheath polymer, i.e., the phase separation during spinning is incomplete. This incomplete phase separation means that some reactive monomers remain in the polymer sheath, where they act as a semi-flexible plasticizer, causing a significant reduction in the $T_{g}$ of the polymer. This then affects the thermal response of the composite fibers, even when the sheath is PVP, normally with very high $T_{g}$.

We attribute the lack of back relaxation upon cooling, seen in the LCE-PVP as well as in the LCE-PLA fibers, primarily due to the presence of the sheath polymer. Because the sheath polymer softens on heating above $T_{g}$, it allows the LCE to contract. Moreover, with the sheath polymer entering a liquid state, we may expect capillary forces at play between adjacent fibers as well as between fibers and a substrate in contact with the fibers. The latter effect is very likely the reason for the frequent partial merging of the fibers upon heating. When we cool back towards room temperature, the sheath polymer stiffens as it goes back into a glassy state, thereby preventing the back relaxation of the enclosed LCE.

We anticipate that a reversible actuation may be achieved if the sheath polymer is removed, but this requires absolute continuity of the core. We have attempted this multiple times by immersing a frame in which free-standing core-sheath fibers are suspended, into a liquid that is a solvent for the sheath but not for the LCE. Unfortunately, in all cases, the fibers are lost. Even tiny discontinuities in the core will lead to a loss of the very thin fibers from the frame once the sheath is dissolved, hence perfect core continuity is a critical requirement, that is challenging to achieve with electrospinning. Better results may possibly be obtained by reverting to a coaxial dual-channel spinneret which separates core and sheath fluids from the beginning.

Finally, it should be stressed that the end-on side-chain LCE geometry developed by the current RM and CL combination is not ideal for LCE actuation. We are in the process of spinning fibers with side-on side-chain LCE as well as main-chain LCE cores, where the stronger actuation of the LCE may give rise to a stronger mechanical response.

## 3. Materials and Methods

### 3.1. Materials and Solution Preparation

Polylactic acid (PLA, *M*${}_{w}$ = 136 kg/mol) is purchased from Jamplast, Inc. (Ellisville, MO, USA). Polyvinylpyrrolidone (PVP, *M*${}_{w}$ = 1300 kg/mol), photo-initiator (PI): 2,2-dimethoxy-2-phenylacetophonone are acquired from Sigma-Aldrich (St. Louis, MO, USA) . The reactive monomer (M): 4-(6-Acryloxy-hex-1-yl-oxy)phenyl 4-(hexyloxy)benzoate is purchased from Synthon Chemicals GmbH (Bitterfeld-Wolfen, Germany) and the crosslinker (CL): 1,4-bis- [4-(3-acryloyloxypropyloxy)benzoyloxy]-2-methylbenzene is purchased from Merck KGa (Darmstadt, Germany). All the solvents used are of GC purity (≥99.8%) sourced from VWR (Merck Millipore). The solvents are dried on molecular sieves (4 Å) overnight to remove the water content and filtered through a polytetrafuoroethylene (PTFE) syringe filter (0.2 μm) or a cellulose acetate syringe filter (1.2 μm) before using. All the handling of reactive components and electrospinning work is done in a yellow room to avoid any pre-polymerization.

In all the reactive mesogen mixture (RMM)-polymer solutions, RMM consists of 86 wt. % of M, 10 wt. % of CL and 4 wt. % of PI. All the spinning solution are stirred at room temperature for 12 h to obtain a homogeneous solution. For PVP fibers, PVP is dissolved in ethanol to obtain a 12.5 wt. % solution. For LCE-PVP fibers, PVP, RMM and a two-solvent mixture [ethanol:chloroform, 8:2 (*v:v*)] with composition [11:10:79, wt. %] are stirred at room temperature for 12 h to obtain a homogeneous solution.

All PLA solutions are mixed in a two-solvent mixture of chloroform and acetone [3:1 (*v:v*)]. For pure PLA fibers, PLA is dissolved in solvent mixture at four concentrations (5, 8, 9 and 10.3 wt. %). In case of CL-PLA fibers three compositions of PLA, CL and solvent mixture as summarized in Table 1 are used. For LCE-PLA fibers spinning solution with composition PLA, RMM and solvent mixture [10.1:10.5:79.4, wt. %] is used.

### 3.2. Fiber Preparation

We have used a home-built electrospinning set-up; more details about the set-up, spinneret and collector design are described in our previous work [20]. Briefly, the electrospinning set-up consists of a nylon stationary stand to which a bare uncoated copper wire (0.38 mm thickness) is attached to serve as the ground electrode, a large insulating chamber (acrylic), a microfluidics pressure control unit (Fluigent, Le Kremlin-Bicêtre, France, model MFCS-EZ, maximum pressure: 1034 mbar), a high-voltage power supply (Gamma High Voltage, model ES30P-5 W/DAM), a digital thermo-hygrometer to monitor the relative humidity during experiments (TFA Dostmann, model 30.5002) and a digital camera to observe the spinning process (Zarbeco, Randolph, NJ, USA, model Z505-OR2) attached to a macro lens (Nikon DGII pro, Tokyo, Japan). The electrospinning set up is placed in a fume-hood. For all the spinning experiments with PVP, relative humidity and temperature are in the range of 30–36% and 18–23 ${}^{\circ }$C, respectively. In case of all the spinning experiments with PLA, relative humidity, and temperature are in the range of 30–60% and 18–23 ${}^{\circ }$C, respectively. The relative humidity and temperature values reported are as measured in the lab during experiments.

The spinning solution is fed through a home-built spinneret consisting of a stainless steel tube (Unimed, Lausanne, Switzerland; inner diameter (ID): 0.70 mm, outer diameter (OD): 1.10 mm, length: 50 mm) with one end connected to a PTFE tube (ID: 0.8 mm, OD: 1 mm) allowing the polymer solution to be flown into the metal tube. The stainless steel tube serves as the high-voltage electrode during spinning. The PTFE tube is inserted into a vial of spinning solution with an 18 gauge, 2${}^{\prime \prime }$ long (18G × 2${}^{\prime \prime }$) bevelled tip syringe needle. Additionally, a short syringe needle is also is connected, via a PTFE tube, to the Fluigent MFCS unit that controls the flow of spinning solution.

The PVP-RMM system is electrospun between 5–8 kV with a flow rate of 7.6 mL/h and a needle-collector distance of 10–12 cm. The PLA-RMM system is electrospun at 7–8 kV with a flow rate of 2.4 mL/h and a needle-collector distance of 15 cm. In all the experiments, fibers are collected on glass slides (Duran, 76 × 26 mm${}^{2}$, Carl Roth, Karlsruhe, Germany) for POM and silica substrates for SEM (SPI Supplies, West Chester, PA, USA, 5 × 7 mm${}^{2}$, #4137SC-AB). For free-hanging aligned samples, fibers are collected between two parallel grounded electrodes and transferred to a frame made of Bytac PTFE surface protection laminate (Sigma-Aldrich, Z278793-1EA).

As-spun fibers are heated to 55 ${}^{\circ }$C on a hot plate so that the RMM in the core of fiber are in the nematic phase and photo cross-linked at 55 ${}^{\circ }$C using a UVATA LED UV curing system (peak wavelength 370 nm, full power intensity 8800 mW·cm${}^{-2}$, Shanghai, China) for 30 min. The system is equipped with four optical fiber heads, which are held 5 cm from the samples.

### 3.3. Fiber Characterization

The optical and morphological properties of fibers are analyzed by polarizing optical microscopy (POM) and scanning electron microscopy (SEM). POM is done in transmission mode using an Olympus BX51 equipped with a Linkam (T95 series LTS120E, Surrey, UK) heating/cooling stage, an Olympus DP73 camera (Tokyo, Japan) and a Canon EOS760D camera (Tokyo, Japan). SEM imaging of the fibers are done using JEOL JSM-6010LA (Akishima, Japan) being operated in 12–15 kV range using an In-lens secondary electron detector. For SEM imaging fiber samples are gold coated (≈25 nm thickness) using a sputter coater (Balzers SCD 050 Sputter Coater) for 100 s. SEM image analysis is done using ImageJ^®^ software to calculate the fiber diameter [42]. The average fiber diameter is calculated by analyzing 50 fibers from different sample locations. Infrared spectroscopy (IR) is done using a Thermo Scientific^TM^ Nicolet^TM^ iS5 spectrometer with id5 ATR accessory.

## 4. Conclusions

We have shown that the electrospinning of a single solution of a supportive high molar mass polymer and an LCE precursor solution can be used to produce LCE core polymer sheath composite fibers, the core-sheath structure forming in situ by spontaneous phase separation and the polymerization and crosslinking to form LCE in the core being initiated by UV irradiation after spinning. The phase separation appears not to be complete, however, as indicated by a glass transition temperature of the sheath polymer that seems to be greatly reduced compared to the pure polymers investigated, as well as by a clearing temperature in the fibers that is much lower than in flat LCE films made from the same precursors. These observations suggest that mesogens are present in the sheath polymer, where they act as a plasticizer. The method yields well-aligned fibers with PVP as well as with PLA as the sheath polymer, and we see a dual response to heating in both cases: the fibers start moving, merging to some extent, and the birefringence reduces. Upon cooling, the birefringence is regained but the fibers do not relax back to their original configuration, hence, the observed actuation is irreversible. We attribute this primarily to the presence of the sheath polymer, which gets soft upon heating above $T_{g}$ but stiffens when the fibers are cooled back, thereby allowing the LCE to respond to heating but not to cooling. These problems are expected to disappear if the sheath can be removed, but to that end, it is very important to have a continuous LCE core. Thus, methods like coaxial dual-channel electrospinning or microfluidics will be tested to make LCE fibers with a thicker core and better definition of the core-sheath interface.

## Figures and Tables

**Table 1:** Composition of PLA and RMM solutions.

Mixture	Components	Composition [wt. %]
1	PLA:RMM:(chloroform:acetone, 3:1)	8:10.5:81.5
2	PLA:RMM:(chloroform:acetone, 3:1)	10.1:10.5:79.4
3	PLA:RMM:(chloroform:acetone, 3:1)	12.2:10.5:77.3

## Supplementary Materials

The following are available online at http://www.mdpi.com/1996-1944/11/3/393/s1, Video S1: Heating of LCE-PVP fibers and Video S2: Heating of LCE-PLA fibers.
